# Supplementary material for: In smiles we trust? Smiling in the context of antisocial and borderline personality pathology
Source: PLoS One. 2020 Jun 25;15(6):e0234574. doi: 10.1371/journal.pone.0234574 (PMC7316324; doi:10.1371/journal.pone.0234574)
Supplement: Attachment — Submitted filename: Response to reviewers - 5-26-2020.docx [file pone.0234574.s002.docx]

May 26, 2020

Dr. Robert Didden

Academic Editor

PLOS ONE

Dear Dr. Didden,

We are delighted to hear that the revisions to our manuscript, “In Smiles We Trust? Smiling in the context of antisocial and borderline personality pathology” (PONE-D-20-06257) improved the paper. We have added the revised figures to include error bars to the current revision.

We thank you again for your helpful comments and suggestions and look forward to hearing from you in due course.

Lawrence Ian Reed, Ph.D.

Ashley K. Meyer

Sara J. Okun

Cheryl K. Best

Jill M. Hooley
